# Nutrition and Food Literacy: Framing the Challenges to Health Communication

**DOI:** 10.3390/nu15224708

**Published:** 2023-11-07

**Authors:** Paula Silva, Rita Araújo, Felisbela Lopes, Sumantra Ray

**Affiliations:** 1 Laboratory of Histology and Embryology, Department of Microscopy, School of Medicine and Biomedical Sciences (ICBAS), University of Porto (U.Porto), Rua Jorge Viterbo Ferreira 228, 4050-313 Porto, Portugal; 2 iNOVA Media Lab, ICNOVA-NOVA Institute of Communication, NOVA School of Social Sciences and Humanities, Universidade NOVA de Lisboa, 1069-061 Lisbon, Portugal; 3 Departamento de Artes e Humanidades, Escola Superior de Comunicação, Administração e Turismo, Instituto Politécnico de Bragança, Campus do Cruzeiro—Avenida 25 de Abril, Cruzeiro, Lote 2, Apartado 128, 5370-202 Mirandela, Portugal; rita.manso.araujo@gmail.com; 4 Centro de Estudos de Comunicação e Sociedade, Instituto de Ciências Sociais, Universidade do Minho, Campus de Gualtar, 4710-057 Braga, Portugal; felisbela@ics.uminho.pt; 5 NNEdPro Global Institute for Food, Nutrition & Health, Cambridge CB4 0WS, UK; s.ray@nnedpro.org.uk; 6 School of Biomedical Sciences, Ulster University at Coleraine, Coleraine BT52 1SA, UK; 7 Fitzwilliam College, University of Cambridge, Cambridge CB3 0DG, UK

**Keywords:** health communication, health policy, nutrition and diet, nutritionist, disinformation

## Abstract

Nutrition and food literacy are two important concepts that are often used interchangeably, but they are not synonymous. Nutrition refers to the study of how food affects the body, while food literacy refers to the knowledge, skills, and attitudes necessary to make informed decisions about food and its impact on health. Despite the growing awareness of the importance of food literacy, food illiteracy remains a global issue, affecting people of all ages, backgrounds, and socioeconomic status. Food illiteracy has serious health implications as it contributes to health inequities, particularly among vulnerable populations. In addition, food literacy is a complex and multidisciplinary field, and there are numerous challenges to health communication that must be addressed to effectively promote food literacy and improve health outcomes. Addressing food illiteracy and the challenges to health communication is essential to promote health equity and improve health outcomes for all populations.

## 1. Introduction

The world is becoming increasingly complex. It is hard to keep up with everything that is going on in the news, let alone all the information needed to know just to navigate your day-to-day life. Every day, people worldwide feel confused about what to eat for health. People look for science-based information but struggle with the vast amount of information and do not know what to believe. People feel that scientists do not agree with each other and are constantly changing their minds. With so much information about what people should eat and how much exercise they should do each day, it can be difficult for even the most educated people to sort through it all, and this confusion can lead them to make unhealthy choices when they are not aware of what they are doing [1,2]. In the first part of the paper, we attempted to clarify the differences between nutrition and food literacy, which are often wrongly used as synonyms. We explored food illiteracy as a major global issue that leads to poor health outcomes and even death [3]. Food illiteracy also promotes health inequities, especially if it is combined with other factors such as poverty and a lack of access to fresh foods or proper nutrition education programs [4]. Food literacy is an important aspect of health literacy because it helps individuals make informed decisions about what they eat and how it affects their health [2]. In a world where processed and fast foods are often more convenient and accessible than nutritious options [5], having food literacy can make a big difference in a person’s overall health and well-being. It can also help to prevent chronic health conditions such as obesity, heart disease, and type 2 diabetes, which are often related to poor diet and nutrition [6]. In the last part of the paper, we explain how food literacy must be framed in the context of health literacy, the importance of both knowledge and skills in food and nutrition, and the broader understanding of health and wellness that is necessary for individuals to make informed choices and decisions about their health.

## 2. Methods

We conducted a literature review to provide an overview of the challenges to health communication concerning nutrition and food literacy. The results are presented as a topical review. We would like to emphasize that this manuscript constitutes a literature review that offers a comprehensive summary of the research findings gathered from diverse sources. Its purpose is to present a holistic overview of existing research on a specific topic without strictly adhering to rigorous systematic review methodologies, such as a formal systematic search strategy or strict inclusion and exclusion criteria. Scopus and Web of Science were used to conduct the search. Multiple search terms were used, including “nutrition literacy”, “food literacy”, “nutritional literacy”, “health literacy”, “health communication”, and “science communication”. Only reports written in English were considered, regardless of whether they were published. A strategy known as “pearl-fishing” or “chaining” chaining’ was employed to identify relevant research articles. This involved a thorough examination of references in other articles, as well as recent articles that cited older, pertinent articles. The selection was limited to papers published in English in peer-reviewed journals and book chapters. Initially, the abstracts were reviewed to gauge their relevance, resulting in the identification of 215 papers. Each of these studies was meticulously studied and analyzed. This process led to the discovery of additional pertinent references, resulting in the inclusion of 161 additional relevant papers in this comprehensive review.

## 3. Health, Nutrition, and Food Literacy

According to Scopus and Web of Science, nutrition literacy appeared first in the title of a scientific document in 1995 in a study carried out by Sullivan and Gottschall-Pass that was carried out to assess the food label nutrition knowledge of healthy Canadians, i.e., to evaluate their nutrition literacy [7]. Nutrition literacy continued to be used without a specific definition; it was used under the umbrella of the more general term health literacy. The definition of nutrition literacy evolved from the tripartite model of health literacy developed by Don Nutbeam, who defined it as a set of cognitive and social abilities that affect an individual’s motivation and capacity to access, understand, and manage information that allows the promotion and maintenance of a healthy status. According to this definition, health literacy is more than being able to read package inserts or being successful in appointments and exam schedules, rather it is being able to make informed decisions [8].

Don Nutbeam [8] identified three health literacy levels: functional, communicative/interactive, and critical. A functional or basic level of health literacy implies knowing how to read and write, understanding simple health messages, and being able to act according to the health information provided. The functional level highlights the importance of knowing health risks, health services, and a set of healthcare recommendations. This includes understanding the labelling of prescription drugs and the ability to read instructions for taking prescription medication. This information is usually distributed to the public through leaflets as the objective is to obtain global benefits [8]. The communicative/interactive level involves a more advanced cognitive level and developed skills that allow individuals to seek and use health information to respond to changing needs. At this level, the focus is on improving individual capacities such as lifestyle changes and the effective use of health services. It also involves the ability to discuss information with health professionals to make informed decisions [8]. For example, it includes understanding a treatment option and how that treatment compares with other options. The most advanced level is critical literacy, which implies an even more advanced cognitive level that allows the individual to critically analyze health information and use the results of their analysis to be alert and thus acquire control over their life events [8]. However, the definition of these three levels of health literacy has some limitations. They only apply to literate communities and assume that a high level of education corresponds to a high level of health literacy and that this is a prerequisite or guarantee that the person will respond in the desired way, which may not correspond to reality [9].

Nutrition and food literacy are two linked concepts related to the ability to understand and apply knowledge about food. Understanding the differences between these concepts is crucial. Definitions in the literature are presented in Table 1. Briefly, nutrition literacy (sometimes mentioned as nutritional literacy) has to do with understanding the role of various nutrients in healthy eating, as well as how to read nutrition labels and make healthy food choices. Food literacy focuses more on the social aspects of food: how it is produced, where it comes from, who grows it, and how these things affect our health.

Nutrition literacy is the level to which people can acquire, process, and comprehend the fundamental nutritional data and services that they need to make correct dietary decisions [10]. This implies having the knowledge of nutritional principles and the ability to understand, analyze, and use nutritional information; that is, to know the nutrients and their health effects [11]. It involves an individual’s capacity to acquire, understand, and use nutritional information from several sources [12]. This includes knowing how foods are digested, their relationship with health, and how to use this information to make healthy choices.

Having nutrition literacy may not be sufficient to achieve the desired well-being and health. It is necessary to have food literacy; that is, to have knowledge, skills, and behaviors that are interrelated and that are necessary to decide, handle, choose, cook, and eat food [13,14,15]. Food literacy is an individual’s ability to make decisions that lead to better individual health status and lead to a sustainable food system considering all social, environmental, cultural, economic, and political factors [16]. Krause et al. (2016) defined nutrition literacy as a subfield of food, with both being specific dimensions of health literacy [17]. According to the authors, nutrition and food literacy are different but complementary concepts. The main difference lies in the skills needed to be literate in nutrition, food, or health. Thus, nutrition literacy consists of the ability to understand basic nutritional information, which is a requirement for a broader array of skills defined for food literacy. The authors suggest the use of food literacy instead of nutrition literacy as it is broader and includes the skills necessary for healthy and responsible eating behavior [17].

Vidgen (2016) identified eight domains of food literacy: (1) access; (2) management and planning; (3) selection; (4) knowledge of food origin; (5) preparation; (6) eating; (7) nutrition; and (8) language [18]. Truman et al. (2017) expanded the components of the definition of food literacy by considering six core themes: (1) capabilities and behaviors; (2) healthy food and choices; (3) culture; (4) knowledge; (5) emotions; and (6) food systems [15].

Food literacy measurement involves evaluating the following skills: reading, understanding, and analyzing information; gathering and sharing nutrition and food knowledge; shopping and preparing food; and evaluating the factors that impact their individual food choices and their influence on society [17].

Vettori et al. (2019) highlight the importance of evaluating the skills needed to access and adhere to a healthy diet when measuring nutrition and food literacy [19]. A nutrition and food-literate community includes people who eat to ensure their health and well-being while ensuring a sustainable food system. Nutrition and food literacy reflect the individual’s inspiration to adopt suitable behaviors and healthy food choices for oneself, others, and the environment [19].

**Figure 1:**
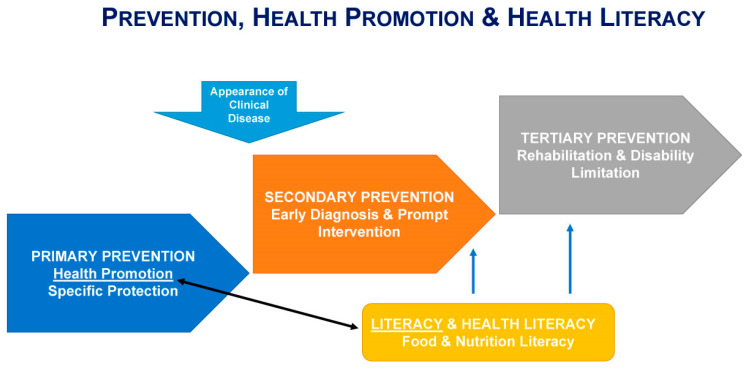
Health, nutrition, and food literacy play a critical role in the prevention and management of diseases across all stages of prevention. The authors acknowledge NNEdPro for the figure conceptualization.

Health, food, and nutrition literacy are critical factors that impact every level of prevention in health and disease (Figure 1). Primary prevention refers to actions taken to prevent the disease onset. Health literacy is essential in promoting healthy lifestyles and behaviors that can prevent chronic diseases. Eating a healthy diet rich in fruits, vegetables, whole grains, and lean proteins can reduce the risk of developing conditions like heart disease, diabetes, and certain types of cancer. Education about the importance of a balanced diet and how to read nutrition labels can also help people make healthier food choices [20,21]. Secondary prevention refers to actions taken to detect and treat a disease early before it causes significant harm. Food and nutrition literacy is critical in secondary prevention because it enables individuals to make informed decisions about their diet and lifestyle to manage chronic conditions such as high blood pressure, high cholesterol, and diabetes. For example, individuals with food and nutrition literacy may be able to modify their diet to reduce their risk of developing complications from these conditions [20]. Finally, tertiary prevention focuses on managing the complications and symptoms of chronic diseases. Health literacy is essential in tertiary prevention because it enables individuals to understand their condition and adhere to their treatment plan. Education about how to read food labels, plan meals, and cook healthy foods can help these individuals better manage their condition and prevent complications [20].

A comprehensive understanding of nutrition literacy is imperative in today’s complex world. It should not be isolated from the two other critical components of health and food literacy. This integration is of paramount importance in providing individuals with a comprehensive understanding of how their dietary choices affect their overall health and broader societal and economic well-being. Health literacy emphasizes the importance of informed decision making in health matters. Food literacy extends health literacy scope to include the social, cultural, economic, and environmental dimensions of food. This broader understanding of nutrition literacy extends beyond simply acquiring knowledge and interpreting food labels. It serves as a reminder that nutrition literacy encompasses a wider social, cultural, and political context, operating at personal, interpersonal, and societal levels. The inclusion of these elements in nutrition literacy helps individuals make healthier food choices and appreciates the wider implications of their dietary decisions. Therefore, a comprehensive conception of nutrition literacy ought to incorporate crucial components of both health and food literacy to provide individuals with a comprehensive understanding of the significance of nutrition in their lives. It is not simply a matter of knowing what to consume; it also involves understanding the rationale behind its significance and integration into the broader context of health and sustainability. This comprehensive approach has the potential to lead to healthy individuals and a sustainable global community [12].

## 4. The Emergence of Food Illiteracy as a Global Issue

The increase in non-communicable diseases related to unhealthy lifestyle habits, including diet, is usually related to food illiteracy increase [22,23]. The latter could be explained by the lack of knowledge about navigating the complex food system and ensuring regular food intake according to nutritional recommendations [19]. Food systems have become highly complex in the 21st century with the “nutrition transition”, characterized by the increase in variety, availability, and accessibility of processed and ultra-processed and low nutrient and energy-dense food [24,25,26]. This type of food is typically mass-produced and has a long shelf life. In addition, it is a very popular food type because it is heavily marketed and easily available in supermarkets, restaurants, and vending machines, or because it can be ordered on the Internet and consumed at home. Today, people have less time to prepare meals from scratch than ever before, which can lead them to consume unhealthy foods that are easy to prepare. The consumption of pre-prepared food and the increase in takeout meals reduces the time spent in the kitchen. Consequently, a decline in cooking and food preparation skills has been observed. As older people stop cooking, they miss the opportunity to teach younger people [24,26,27].

The emergence of food illiteracy as a global issue due to the modern lifestyle and diet of many people around the world is a serious problem [24]. Several factors contribute to food illiteracy, including a lack of access to education about healthy eating, lack of access to affordable healthy food, lack of time to cook healthy meals, lack of knowledge about how to prepare healthy meals, and high levels of stress, which can lead to unhealthy eating habits [28]. These determinants of health play a significant role in shaping food and health literacy [17]. They are crucial for governments when determining equity and resource allocations. Therefore, directing more funding towards increasing literacy, especially among disadvantaged groups, can be a key strategy in addressing the broader issue of food illiteracy and its associated health challenges.

Food illiteracy is a growing problem in many countries worldwide as people are exposed to misinformation about the food they consume and its effects on their health [29]. When people have a low level of food literacy, they may be unaware of how much salt or sugar is in their food or how many calories are in a serving. They may not know which ingredients are good or bad for them. They might not realize what types of foods they should eat more often or less often, based on their age or health status [30,31,32]. There are two types of food illiteracy: the first is when people do not know what certain foods are or how to prepare them, and the second is when people have a poor understanding of the nutritional value of different foods and do not know how to properly balance meals [28]. This lack of knowledge about what we eat has led to unprecedented rates of obesity and diabetes, which often lead to other health problems such as cardiovascular disease and kidney disease. Although many people know that diet plays a role in weight gain and obesity, they may not realize that diet also has an impact on diabetes [33]. Research has suggested that certain dietary habits can help reverse diabetes by enhancing insulin sensitivity and reducing blood sugar levels [34]. If the prevalence of obesity continues to increase, almost half of the world’s adult population will become overweight or obese by 2030 [35]. The government and health authorities have highlighted the health and social complications related to inappropriate dietary patterns [36].

Children’s food literacy rates are low. Many schools or day-cares do not provide students with healthy meals during school hours; instead, they rely on parents to pack lunches from home or provide snacks from vending machines that only offer sugary snacks such as chips or candy bars instead of healthier options such as fruits or yoghurt bars [37]. Early life stages represent an important period since lifelong habits are developed and life circumstances influence health outcomes in adulthood. The important social, emotional, and cognitive changes that occur during youth mean that there is a greater tendency to engage in risky behaviors during this life phase. Irresponsible eating habits can have negative health effects with individual, familial, and social consequences [38]. Therefore, improving healthy eating habits and lifestyles in the early stages of life is of major concern worldwide. Eating is a socially constructed practice that is important for understanding and pursuing health [39]. However, food and eating practices occupy a relatively minor place in the scholarship. Communication with the public is the initial stage of the behavior-change process and is a crucial piece of effective public policy [40]. To combat this problem, governments should invest more money in educating children about healthy eating habits at school so that they can make good choices throughout their lives [41].

Food illiteracy has also been shown to be a major contributor to food waste. Approximately one-third of all food produced worldwide is wasted, and this number continues to increase annually [42]. This is a problem because of wasted resources and can have serious health, social, economic, and environmental effects [43]. Food waste begins at home, where consumers often buy more than they need or want and then waste what is left over when they do so [44]. The health effects of food waste are numerous. For example, the loss of nutrients from discarded fruits and vegetables means that people who eat this food have a lower nutritional intake than those who do not [45]. Additionally, when food is discarded instead of eaten, it causes greenhouse gas emissions by rotting in landfills [46,47]. Food waste also has economic and environmental effects. If more fruits and vegetables are eaten rather than thrown away after reaching their expiration dates, more money would be saved by consumers who would not need to buy new produce. Moreover, people consume more processed foods than ever before, causing them to throw away more packaging [48]. However, there are other factors at play here too: climate change is causing droughts around the world, which means that crops are failing due to a lack of rain and heat stress on plants during ripening periods. The result is less food available for everyone, from farmers down to consumers, which means higher prices on produce at grocery stores which causes consumers to buy less healthy foods overall because they are too expensive for their budgets [49].

Measuring food literacy is of the utmost importance. According to a recent scoping review, there are now 12 tools available to assess adult food literacy. The authors found that even though most of them cover all four aspects of food literacy, theoretical frameworks are rarely applied. They also concluded that existing tools have only been tested in a few situations, and they mainly rely on biased self-reporting techniques [50]. The self-perceived food literacy scale (SPFL) and the short food literacy questionnaire (SFLQ) are the ones that have been validated in more countries [51,52,53,54,55,56]. Both primarily concentrate on individuals’ skills and competencies needed for making appropriate food choices [57]. The SPFL scale was created by Poelman et al. (2018) to measure adults’ food literacy concerning healthy diets, knowledge, skills, and behaviors necessary for the appropriate planning, management, selection, preparation, and consumption of food [53]. Despite limited application, generally, SPFL revealed that better food literacy was linked to better impulse control, less impulsive behavior, and healthier food consumption [53] and that adults with a low and medium level of education have a low food literacy level [58]. The relationship between Body Mass Index (BMI) and SPFL was also analyzed, and depending on the context, BMI may predict [52,59] or have no effect on the SPFL final score [58].

The SFLQ [57] scale has been used in food and nutrition intervention research to assess respondents’ functional, interactive, and critical food literacy abilities. Consumers in Italy who had received specialized education in human nutrition scored higher on the SFLQ and SPFL than those without this knowledge. Furthermore, the SFLQ scores were notably higher among women [59].

## 5. Food Literacy Health Inequities

In recent years, there has been an increased interest in exploring the relationship between food literacy and health outcomes. As already mentioned, people who lack basic knowledge about food tend to be more likely to develop illnesses like obesity, heart disease, diabetes, and cancer [6]. Therefore, it is important to understand the factors that contribute to the issues surrounding food literacy and health inequities to help policymakers and educators combat them altogether. Sometimes, inequity and inequality are wrongly used as synonyms, although the former entails determining whether the inequality is morally incorrect, while the latter is just a dimensional description used anytime numbers are uneven [60]. “Health inequities are differences in health status or the distribution of health resources between different population groups, arising from the social conditions in which people are born, grow, live, work and age” [61]. These social factors may include, but are not limited to, democratic decision-making procedures, accessibility of financial resources, environmental disadvantages, and opportunity structures.

Health inequity is evident across all life stages, from birth to old age [62]. Hargrove (2018) found that BMI trajectory throughout the transition to adulthood is shaped by several factors, including race and ethnicity, gender, and socioeconomic level of origin [63]. A recent retrospective study found that early life adversity was associated with overall dietary quality outcomes. However, the authors suggested that other factors also drive dietary quality, including limited access to reliable information about food to make informed decisions about food choices for healthy living [64]. These studies show that the relationship between health and socioeconomic status is not simple, nor does it adhere to a single linear pattern. Rather, health disparities are influenced by multiple factors that may reflect a complex combination of the effects of social disadvantage, discrimination, power differentials, and access to resources within environments that include cultural norms, policies, and practices. Butcher et al. (2020) conducted a case study to analyze Australian food literacy education programs throughout the life cycle and their effects on reducing health disparities for those who are at risk of social and economic disadvantages. The authors found that programs promoting food literacy are essential to enhance the health of vulnerable groups [65].

Food literacy is especially important for disadvantaged populations as they are more likely to suffer from food-related health inequities due to their limited access to healthy foods and information about how healthy diets can improve their lives. It is noted that most people in developed nations have high levels of food literacy, but those in developing countries do not [66]. Even in industrialized nations, those who live in rural and regional areas are more likely to face social and economic deprivation, prejudice, and possible social or physical isolation; all these factors may eventually affect their access to health care and wholesome food [65,67]. Moreover, marketers of unhealthy food usually target disadvantaged populations that have fewer opportunities to correct disinformation [68,69,70].

Education programs should focus on improving levels of food literacy among populations with low levels of education—this would help reduce health inequities globally while also making sure that everyone has access to nutritious foods and knows how best to prepare them.

## 6. Food and Nutrition Disinformation: Challenges to Health Communication

Media and personal experiences influence individual food choices. The information consumed about a specific food impacts the individual perception of it. Food and nutrition disinformation is one of the challenges to health communication. Disinformation spread through various channels, such as social media, online forums, and even mainstream media, can lead to confusion and misinterpretation of nutritional information, resulting in harmful dietary practices and misinformed health decisions. It is crucial to conduct a critical analysis of the information and to seek out credible sources for nutrition advice [71,72].

The internet and social media enable information to be shared rapidly and widely, including disinformation. Therefore, it is difficult for health authorities to counter false claims and keep up with untrue information spread. There are several reasons why people may turn to the Internet and social media for food and nutrition information [73]. Some people may be looking for specific information about a type of food, such as nutritional values or health benefits [74]. Others may be looking for recipes, meal ideas, or cooking tips [75]. Additionally, some people may be motivated to use the Internet and social media for food and nutrition information due to health concerns, such as weight loss, diabetes, or food allergies [76,77]. Social media can also play a role in people’s food choices and food-related behaviors as they may be influenced by the food-related content they see on these platforms [78].

Some food and beverage companies, as well as individuals and organizations, spread disinformation to promote their financial interests. Disinformation about food and nutrition is spread by the food industry through advertising, marketing, and lobbying, which can contribute to confusion and misconceptions about food choices [79]. This can include promoting unhealthy products or claiming that certain ingredients are more healthy than they are [1]. There is evidence that food advertisements go against nutritional recommendations, which blocks the flow of knowledge about nutrition [69,80].

Many people lack the scientific literacy needed to critically evaluate nutritional information and distinguish between credible and non-credible sources [81]. Despite the individual level of literacy, there is often a lot of conflicting information about what is considered a healthy diet, making it difficult for people to determine what is truly beneficial for their health [82]. A recent study found that despite Mexican American women articulating links between diet and disease, they felt overwhelmed and perplexed by the contradicting information from both official and private sources. Moreover, those women mentioned the existence of critical information gaps, distinguishing between the information available and the information needed [83]. Multiple and conflicting sources of information are also something that parents usually face when trying to provide healthy food [84].

Despite the efforts of government agencies and the existence of healthy eating guidelines, there is an ongoing need to address these challenges. Health organizations must continue to provide credible, evidence-based information and promote scientific literacy among the public. Health professionals and nutrition experts should be vigilant in detecting and countering misinformation regarding food and nutrition. Furthermore, social media companies and other online platforms can play a pivotal role in reducing the spread of disinformation by promoting credible sources of information and limiting false claims.

### 6.1. Health Communication

Before defining nutrition and food communication, it is also important to clarify the meaning of health communication. Simply, it is the application of communication approaches to inform and affect decisions and actions to improve individual and global health. However, this simple definition has changed over time.

In 1996, Roger defined health communication as any human communication type related to health [85]. In the same year, Ratzan, Payne, and Bishop advanced with a more complete definition by affirming that health communication deals with the combination of a man’s physical, occupational, and intellectual dimensions, which guarantee human well-being [86]. Two years later, Gary Kreps and his colleagues (1998) described health communication as a vibrating and critical field of study dealing with the influence of human and mediated communication in health care delivery and health promotion [87]. In the same article, the authors distinguish health care delivery and health promotion as two sub-areas of health communication study. Researchers dedicated to health care delivery analyze the effects of communication in the provision of health care. Those dedicated to the study of health promotion aim to study the messages and means used in public health promotion [87]. Later, in 2006, the importance of health communication in promoting public health was reinforced by Thomas, who said that health communication is a powerful tool for transmitting messages that impact the quality of life of populations and policy formulation [88]. According to Thomas, communication can be between a physician and patient or a health or communication professional and the public and can use a wide range of print, audio-visual, and virtual media to achieve its goals [88]. In 2009, the book “*Emerging perspectives in health communication: Meaning, culture, and power*” was published, in which the authors, Heather M. Zoller and Mohan J. Dutta, define health communication as a set of communication processes and messages in which health issues are the focus [89]. Zoller and Dutta divided health communication into two sub-areas, which were different from the ones previously established by Kreps et al. [87]: one that studies communication processes and one that analyses messages [89].

According to Liu and Chen (2010), health communication is essential for health prevention and promotion, doctor–patient relationship improvement, public health awareness, and health risks communication [90]. The same authors remarked that health communication must be done through different channels to be successful in influencing people’s attitudes and in encouraging behavior change through a more healthy lifestyle. Liu and Chen argued that successful health communication strengthens an individual’s awareness of the value of their health and improves their readiness to face potential future health challenges. Additionally, Liu and Chen highlighted the importance of communication to change public health, improve public health services, and improve life quality at individual and community levels [90]. Schiavo’s (2013) definition of health communication is one of the most agreed-upon definitions within the scientific community: “health communication is a multifaceted and multidisciplinary field of research, theory, and practice. It is concerned with reaching different populations and groups to exchange health-related information, ideas, and methods in order to influence, engage, empower, and support individuals, communities, health care professionals, patients, policymakers, organizations, special groups and the public, so that they will champion, introduce, adopt, or sustain a health or social behavior, practice, or policy that will ultimately improve individual, community, and public health outcomes.” [91].

### 6.2. Nutrition and Food Communication

Food is a basic and essential need for human survival and healthy life. Eating behavior depends on several factors, including environmental, economic, political, and social [92]. In recent decades, all over the world, there has been an increase in diseases related to unhealthy diets. Inadequate nutrition practices are associated with an increased risk of noncommunicable diseases [93]. Obesity, for example, is one of the major risks for heart failure [94], type 2 diabetes [95], hypertension, and coronary heart disease [96]. In this context, nutrition and food communication is crucial to influence healthy eating behaviors.

The principles for effective nutrition and food communication are, in many ways, close to the theories of social marketing because it applies marketing rules and procedures to persuade a target audience to voluntarily accept, refuse, change, or stop a specific behavior [97]. Kotler and Zaltman (1971) first proposed that the principles of commercial marketing could be applied to ideas, behaviors, and relevant social programs. According to them, social marketing is the design, execution, and control of strategies that seek to increase the approval of a social idea or practice by a specific target audience. Therefore, marketing thinking can be applied to a range of social activities carried out by “non-business organizations” [98].

Andreasen’s definition of social marketing emphasizes that the purpose of social marketing is behavioral change. He defined social marketing as the application of commercial marketing tools to explore, develop, implement, and evaluate programs aimed to induce behavior change to improve individual well-being and that of society at large [99]. This definition includes four concepts: 1) voluntary behavioral change; (2) clear identification of the benefit of behavioral change by the target audience; (3) use of marketing techniques (consumer orientation, market research, segmentation, audience definition and marketing mix); and (4) individual improvement and collective well-being, not of the organizations involved in social action [100].

Social marketing implies a systematic planning process focused on behavior change, the development of clear messages, the design of interventions, their implementation, and the continuous evaluation of results [97]. Social marketing is a tool with great potential to motivate behavior change toward better health and, more specifically, is an effective mode of promoting healthy diets with individual, family, community, and social effects [101,102,103,104].

As with other health topics, food communication occurs at three different levels: at an individual level, studying the effects of communication on food knowledge and behaviors and the influence of interpersonal relationships between people, family members, health professionals, and their respective social networks on health outcomes. It occurs at an organizational level in the study of the effects of public communication on individual health. And finally, it occurs at a societal level, studying the effects of communication on large-scale social changes. Consequently, researchers must carry out multi-level research to identify, criticize, explain, predict, and understand the intrapersonal, interpersonal, group, organizational, cultural, media, and relevant social systems dynamics [105].

The dietary choices made by individuals have a significant impact on their health, which aligns with fundamental principles of public health. To promote public health, it is essential that individuals are equipped with knowledge and resources to comprehend and choose healthier food alternatives. Therefore, improving food communication with the public within the realm of public health initiatives is a pressing priority. This comprehensive communication strategy encompasses the dissemination of crucial messages not only by government agencies and health organizations but also by media outlets and food companies. The implementation of effective food communication, firmly rooted in the principles of public health, plays a pivotal role in enabling individuals to make informed dietary decisions. These decisions, in turn, have a significant impact on overall well-being, aligning with the fundamental objectives of public health initiatives [106].

As for other health topics, food communication must be framed in a way that is effective, trustworthy, and beneficial for public health. Therefore, the information should be accurate, science-based, and free from bias or conflicts of interest. Food communication must be audience-centered, clear, concise, easy to understand, and relevant to the needs and interests of the target audience. Moreover, the information should consider the audience’s cultural, social, and economic background. The information source should be trustworthy and credible, with a reputation for providing accurate and reliable information [107]. The information should empower people to make informed decisions about their food choices and health.

Governmental agencies can play a vital role in increasing food literacy by providing citizens with resources that help them make informed decisions about food. Governments must promote healthy eating habits through policy changes such as taxes on unhealthy foods [108,109,110,111] or subsidies for fresh vegetables and fruit [111]; these policies will help people make healthier choices such as reducing the consumption of salt [112] or sugar [113,114]. According to Nnoaham et al. (2009, p. 1330) “tax on unhealthy foods, combined with the appropriate amount of subsidy on fruits and vegetables, could lead to significant population health gains” [115]. This combination assumes particular importance in low-income populations, where governmental authorities should work with private organizations and businesses to implement programs that increase access to fresh fruits and vegetables [116,117]. While taxes and subsidies can be useful tools in reducing consumption and promoting health, they are not a panacea and must be implemented as part of a comprehensive approach that includes education, awareness-raising, and the availability of healthier options. When evaluating the effectiveness of taxation and subsidy programs, it is crucial to consider scientific research findings related to the factors influencing taxation outcomes. This includes understanding how taxes on unhealthy foods and beverages impact supply side changes in the nutritional value and environmental sustainability of the food supply. Additionally, it involves assessing consumer attitudes and knowledge about food, as well as macroeconomic outcomes such as governmental revenue, consumer welfare, and overall population and planetary health outcomes [110].

Providing clear and concise information about the ingredients, nutritional value, and origin of food products through labelling can help consumers make informed decisions about what they eat. Labels are a powerful tool to communicate nutrition information and improve food literacy since most people use this information consistently. Although a significant part of the population report looking for nutrition information on the label when shopping, they do not understand all of the information contained in the label [118,119]. Some studies report a lack of knowledge about some concepts like energy and energy terms [119], fiber [120], and serving size information [121]. Front-of-package nutrition messaging may guide consumers to healthy choices in some cases, but the understanding of label nutrition information depends on several socio-demographic factors and their interaction. For example, younger adults may be more likely to interpret label nutrition information [122,123,124,125], which does not necessarily mean that they use a food label to the desired level [120], and sometimes this can result in undesired consequences such as impacting young adults’ relationships with food and disordered eating [126]. Regarding gender, there are also some controversial results. Some studies revealed that females are more likely to use nutrition information on labels [118,122,127]. However, others did not find differences between male and female consumers in label nutrition information use [128,129]. Concerning socioeconomic factors, several studies have focused on education as a key indicator of the utilization of labelled nutrition information. People with higher levels of education tend to better understand nutrition information and how to interpret it [122,128,130,131]. Some studies show that low-income individuals may struggle to understand complex nutrition information [122,124,125,128]. These socio-demographic factors can interact and compound, making it difficult for some individuals to understand and use nutrition information effectively. It is important for nutrition education and outreach efforts to take these factors into account to ensure that everyone has the information they need to make informed food choices.

Encouraging people to grow their own food or participate in community farming programs can increase their understanding of where food comes from and how it is produced [132]. Teaching people how to cook healthy and nutritious meals can also increase their food literacy and knowledge of healthy eating habits [133,134]. Findings suggest that the incorporation of cooking programs and food literacy education into the curriculum can help young people develop healthy eating habits and a better understanding of food, which may positively influence children’s food-related preferences, attitudes, and behaviors [135,136,137,138].

Governments worldwide have expressed their concern by funding public education campaigns that promote the importance of food literacy and provide information on healthy eating habits, food preparation, and cooking. Launching these campaigns can significantly increase awareness and understanding among the public. Effective communication about nutrition improves people’s knowledge and ability to make decisions that lead to healthier diets. Regardless of the size, budget, and time available, successful message creation, implementation, and development require the involvement of researchers and multidisciplinary participation [100,139]. Most social marketing campaigns target young people, children, and teenagers and aim to change behaviors. In most cases, the intention is to encourage the consumption of fruit and vegetables and the adoption of healthier practices [140]. The most well-known food and nutrition campaigns, which served as the impetus for subsequent ones, began in the United States. The Henry L. Kaiser Family Foundation introduced “Low-Fat Eating for America Now” in 1987 to lower fat consumption to 30% of total calorie intake [141,142]. The California Department of Health Services began the “5 a Day for a Better Health” program in 1991 to promote the consumption of fruits and vegetables [143]. Also in 1991, the “Energize Your Life!” campaign was launched to promote healthy behaviors such as a balanced diet and physical exercise [144]. Also in the United States, the “Steps” campaign was implemented in 2005, aimed at African American women aged between 18 and 49, which, in addition to recommending the consumption of fruit and vegetables, sought to motivate them to practice regular physical exercise [145]. In 2007, the “5 a Day for a Better Health” campaign was replaced by the “Fruits and Veggies—More Matters” campaign, which aimed to increase adult knowledge about the recommendation and benefits of eating 7 to 13 servings of fruits and vegetables daily [146]. Between 2018 and 2019, there were several phases of the “Celebrate Your Plate health” campaign created by the University of Ohio, which had its pilot year in 2017. The campaign aimed to increase the consumption of fruits and vegetables and used a mix of communication channels, both print and digital. The study that was carried out to evaluate the effects of the campaign concluded that the campaign led to greater consumption and a greater intention to increase the consumption of fruits and vegetables but warned of the need for further studies to be carried out to determine the reason for causality [147]. Still in the United States, specifically in Multnomah County, Oregon, the campaign “It Starts Here” was launched in 2011 to inform residents about the high amount of sugar in soft drinks and sugary juices [148]. That same year, in Philadelphia, the “Get Healthy Philly” campaign was launched, aiming to reduce the consumption of sugary drinks by children to prevent obesity. As part of this campaign, leaflets were distributed and advertisements were made for radio and television. The use of the media was very favorable because, in addition to its great reach, a change of discourse was observed in different contexts directed towards the prevention of obesity [149]. In addition, the campaign increased the number of parents who changed their behavior after seeing the television advertisement and who started giving fewer sugary drinks to their children [150]. In 2016, the “Our health is in our hands” campaign was launched to raise obesity awareness and increase involvement in prevention, nutrition, and exercise programs offered by the Brooklyn Partnership to Drive Down Diabetes (BP3D) [151]. The excessive consumption of salt is also a concern of the authorities because it contributes to the onset of chronic diseases. During 2014–2015, the Philadelphia Department of Public Health conducted a campaign through radio, print news, and traffic signs to promote awareness of the link between salt consumption and cardiovascular disease and stroke [152]. Outside the United States, we highlight the campaigns carried out in Europe, such as “La Santé Vient en Mangeant” resulting from the “Manger Bouger” program launched in 2002 in France. The campaign aimed to improve the health status of the French, considering nutrition to be one of the change factors [153]. The campaign “Le Plaisir de Bien Manger et d’Être Actif!” began in 2007 in Luxembourg and consisted of a set of nutritional recommendations [153]. In Italy, the ViviSmart campaign aimed to promote a healthy diet and lifestyle among primary school children [154]. The first Portuguese campaign launched in 2017, under the slogan “Juntos contra o sal”, aimed to reduce salt intake by 3 g per day [155,156] and was followed by the campaigns “Juntos contra o açúcar” and “Juntos pela Alimentação saudável” [155,156]. In 2019, the first Portuguese campaign to promote a healthy diet was massively promoted through different means (television, radio, billboards, public transport, social media, and regional press). This campaign, entitled “Comer melhor, uma receita para a vida”, aimed to encourage the consumption of fruit, vegetables, legumes, and water [155,156].

Using social media and online resources to share information about food, nutrition, and healthy eating can reach a large audience and help to increase food literacy. One of the oldest digital ways of providing nutrition information is through websites. One study showed that interaction with the “5 a Day, the Rio Grande Way” website resulted in increased fruit and vegetable consumption by adult inhabitants of rural areas [157]. Digital interventions need to be audience-specific to be effective, as shown by obesity interventions for low-income mothers in rural areas [158], for teenagers [159], or to encourage healthy eating habits among middle-aged men for the prevention of chronic diseases [160].

The use of digital resources should be encouraged in classrooms. One study analyzed a blog created by students as part of a nutrition course called Food Logging and Blogging. The results show that blogs are an asset for promoting nutrition literacy and that their use in a pedagogical context is important [161]. Online information about healthy eating habits is needed to counterbalance the large number and diversity of marketing campaigns encouraging unhealthy food consumption to which young people are exposed. According to Cairns et al.’s (2013) review, most food marketing strategies target children and are for foods high in fat, salt, or sugar, such as sugary breakfast cereals, fast food, and soft drinks [162]. Rowbotham et al. (2020) conducted a review to map the way chronic disease prevention is addressed by the media and found that nutrition-related issues were predominant and that this issue had been driven by the fact that children are highly exposed to advertisements for unhealthy food [163]. A study carried out among mothers who wrote blogs where they provided information on maternal care, including health, showed that most articles were about nutrition and that these were also the most appreciated by followers. The study alerts us to the importance of these blogs being able to be used for successfully transmitting health information [164].

Nutrition is one of the most covered health topics on social media and this is increasing. A recent study that analyzed the behavior of adolescents concerning health-related content on social media showed that food care was the topic sought by 30% of respondents [165]. A retrospective analysis of the Facebook^®^ pages of German insurance companies also found that healthy eating is one of the topics most discussed on that social media [166]. Marketing campaigns such as Food Hero created by Oregon State University in 2009, within the scope of the Extension Nutrition Education Program, to promote the consumption of vegetables and fruits, were more successful on social media, namely Facebook^®^, Twitter^®^, and Pinterest^®^, than through the other channels used [167]. As with other digital interventions, creators of nutrition content for social media must consider the target audience, the most appropriate platforms, and the most appropriate strategy for each of them [168]. The message and its source are the most important factors to consider. An experimental study analyzed the reaction of 700 secondary school students to images used in an anti-obesity campaign carried out on blogs, digital news, and Facebook^®^. It was found that interaction with the media depends on the credibility of the source and that the campaign affects self-perception of weight, but the authors warn that there is a risk that images with provocative messages will have an effect contrary to the desired one [169]. When social media is used with nutrition messages targeted at young people, healthcare professionals face competition from influencers. A study compared young people’s reactions to Instagram^®^ and Youtube^®^ messages linked by influencers and nutritionists and concluded that the latter must improve their approach to these media [170]. The study recommends that those professionals use social media to inform young people and that they should create attractive and impactful messages. It also recommends that professionals report their own experiences and life stories, which their followers identify as increasing authenticity and reliability and generate greater persuasiveness [171]. An analysis on Twitter^®^ confirmed that, also on this platform, most articles published on nutrition are written by people with no training in the health field and that many of them can produce unwanted results [171]. Another study revealed that during the recent COVID-19 pandemic, nutrition was among the topics associated with a greater number of fake news [172]. On the other hand, the pandemic seems to have alerted nutritionists to the communication potential of social media to increase health literacy and not just to publicize their services, as they had done until then [173]. In short, social media has a high potential as a means of communication and it is important that health professionals [171] and other institutional entities, such as, for example, those responsible for school nutrition programs [2], start using them regularly and effectively.

Communication is a fundamental component of enhancing healthcare quality as it enhances patient safety, ensures care coordination, supports patient-centered practices, and facilitates collaboration among healthcare teams. An effective communication strategy ensures that healthcare providers, including doctors, nurses, specialists, and support staff, are on the same page regarding a patient’s treatment plan. Whenever everyone is informed, there is improved coordination in delivering care, reducing the risk of errors and ensuring that patients receive the right interventions at the right time. Clear and open communication helps to identify potential safety issues and prevent medical errors [174,175]. The provision of patient-centered care requires effective communication. It involves listening to patients, understanding their concerns, and involving them in decision-making regarding their treatment. This approach enhances patient satisfaction and leads to improved health outcomes [176]. Effective communication systems within healthcare organizations allow for the collection and analysis of data related to patient care. By sharing insights and outcomes, health care providers can identify areas where quality improvement is needed and implement changes accordingly [177]. In healthcare settings, various professionals work together as teams to manage patients’ healthcare. Communication among team members fosters collaboration, ensuring that everyone understands their role and responsibility. Miscommunication can result in misunderstandings, delays in care, or misinterpretations of patient information. Open and clear communication helps to prevent these issues, ensuring that healthcare providers receive accurate and relevant information about patients [178]. Communication with the patients is essential for their education. Those who are well informed are more likely to follow their treatment plans and make healthy lifestyle choices. Effective communication channels enable health care professionals to remain informed and actively participate in continuous learning, thereby elevating the standard of care they provide. Patients who feel heard, respected, and informed about their care are more likely to be satisfied with their healthcare experience, which can have a positive impact on the overall quality [175]. Communication is often a legal and ethical requirement in healthcare. Clear communication of risks and benefits is essential to meet these obligations and maintain the highest standards of care. By prioritizing effective communication, healthcare organizations can enhance patient outcomes and provide superior care.

## 7. Conclusions

Nutrition and food literacy are different concepts which refer to the understanding of the role that food plays in maintaining health and well-being. This includes knowledge of the nutritional value of different foods, the impact of food choices on health, and the ability to access, prepare, and cook nutritious meals. Unfortunately, the emergence of nutrition and food illiteracy as a global issue has led to widespread health problems, including the rise of chronic diseases such as obesity, diabetes, and cardiovascular disease. The creation of food literacy and health inequities is also a concern as certain groups, such as low-income populations and marginalized communities, may face additional barriers to accessing healthy food and nutrition information. This can lead to health disparities and further exacerbate existing health inequities. To address these issues, there is a growing need for food literacy pathways to health. This includes education and training programs that provide people with the knowledge, skills, and resources they need to make informed food choices and to access, prepare, and cook nutritious meals. These programs should be culturally and linguistically appropriate and should be accessible to all populations, including those who are at risk of nutrition and food illiteracy. Food and nutrition disinformation is another major challenge to health communication as it can lead people to make unhealthy food choices and undermine efforts to promote healthy eating. This is particularly true in the digital age, where false or misleading information about food and nutrition can spread quickly and widely through social media and other online channels. It is important for health professionals, educators, and policymakers to work together to address these challenges and to promote accurate and trustworthy information about food and nutrition. In conclusion, nutrition and food literacy is a critical aspect of maintaining health and well-being, and addressing the global problem of nutrition and food illiteracy is essential for promoting public health. By providing people with the knowledge, skills, and resources they need to make informed food choices and to access, prepare, and cook nutritious meals, we can help to improve health outcomes and reduce health inequities. The future of nutrition and food literacy holds immense promise. The complexities of the modern world present exciting opportunities for research, education, and policy development. Technological innovations, such as mobile apps and online resources, can be used to provide accessible and personalized nutrition guidance. Collaboration among healthcare professionals, educators, and community leaders can help create comprehensive food literacy programs that empower individuals from all backgrounds to make informed choices regarding their diet. Furthermore, we must advocate for policies that promote equitable access to healthy food and accurate nutritional information, especially for under-served populations. By taking advantage of these opportunities and forging partnerships, we can collectively work towards a future where nutrition and food literacy become powerful tools for improving public health, reducing disparities and fostering a culture of well-informed healthy living.

## Figures and Tables

**Table 1:** Nutrition and food literacy definitions found in the literature.

**Nutrition Literacy Definition**	**Reference**
“Nutritional literacy, as a specific form of health literacy, requires both general literacy and computational skill. It is not surprising to find that higher levels of nutrition knowledge have been associated with nutrition label use.”	Blitstein, J.L.; Evans, W.D. Use of Nutrition Facts Panels among Adults Who Make Household Food Purchasing Decisions. Journal of Nutrition Education and Behavior 2006, 38, 360–364, doi:10.1016/j.jneb.2006.02.009. (p. 1)
“Adequate health literacy and nutrition literacy require an individual not only to read well, but also to understand health and nutrition concepts and to have basic quantitative skills (defined as numeracy: the ability to use and understand numbers in daily life, including the ability to read and interpret nutrition information. People without these skills may have difficulty understanding concepts of healthful diets, reading nutrition information, and measuring a portion size.”	Neuhauser, L.; Rothschild, R.; Rodríguez, F.M. MyPyramid.gov: Assessment of Literacy, Cultural and Linguistic Factors in the USDA Food Pyramid Web Site. Journal of Nutrition Education and Behavior 2007, 39, 219–225, doi:10.1016/j.jneb.2007.03.005. (p. 220)
“Nutrition literacy can be defined similarly to health literacy as the degree to which individuals can obtain, process, and understand the basic health (nutrition) information and services they need to make appropriate health (nutrition) decisions, with the qualification that the definition is nutrition-specific.”	Silk, K.J.; Sherry, J.; Winn, B.; Keesecker, N.; Horodynski, M.A.; Sayir, A. Increasing Nutrition Literacy: Testing the Effectiveness of Print, Web site, and Game Modalities. Journal of Nutrition Education and Behavior 2008, 40, 3–10, doi:10.1016/j.jneb.2007.08.012. (p. 4)
“Nutrition literacy may be defined as the degree to which people have the capacity to obtain, process, and understand basic nutrition information.”	Zoellner, J.; Connell, C.; Bounds, W.; Crook, L.; Yadrick, K. Nutrition literacy status and preferred nutrition communication channels among adults in the lower Mississippi Delta. Preventing Chronic Disease 2009, 6, doi:19755004. (p. 2)
“In other words, at what point is this client no longer dependent on expert knowledge? When do his or her food choices reflect what is right for him or her 80% to 90% of the time? That is when the person achieves nutrition literacy. Fortunately, the term can and should be individualized according to the goals set at the beginning of the relationship. When that individual says, “I can do this on my own”, the dietetics practitioner will have succeeded.”	Escott-Stump, S.A. Our nutrition literacy challenge: Making the 2010 dietary guidelines relevant for consumers. Journal of the American Dietetic Association 2011, 111, 979, doi:10.1016/j.jada.2011.05.024. (p. 979)
“…‘nutrition literacy’ can mean the extent to which people access, understand and use nutrition information. In this context, consumers’ nutrition literacy is critical in their interpretation of noninterpretative front-of-pack food labelling and menu labelling.”	Watson, W.L.; Chapman, K.; King, L.; Kelly, B.; Hughes, C.; Yu Louie, J.C.; Crawford, J.; Gill, T.P. How well do Australian shoppers understand energy terms on food labels? Public Health Nutrition 2013, 16, 409–417, doi:10.1017/s1368980012000900. (p. 410)
“Functional nutrition literacy refers to proficiency in applying basic literacy skills, such as reading and understanding food labelling and grasping the essence of nutrition information guidelines. Interactive nutrition literacy comprises more advanced literacy skills, such as the cognitive and interpersonal communication skills needed to interact appropriately with nutrition counsellors, as well as interest in seeking and applying adequate nutrition information for the purpose of improving one’s nutritional status and behaviour. Critical nutrition literacy refers to being proficient in critically analyzing nutrition information and advice, as well as having the will to participate in actions to address nutritional barriers in personal, social, and global perspectives. CNL is part of scientific literacy– ‘the capacity to use scientific knowledge, to identify questions and to draw evidence-based conclusions’ i.e., proficiency in describing, explaining and predicting scientific phenomena, and understanding the processes of scientific inquiries as well as the premises of scientific evidence and conclusion.”	Guttersrud, O.; Dalane, J.O.; Pettersen, S. Improving measurement in nutrition literacy research using Rasch modelling: Examining construct validity of stage-specific ‘critical nutrition literacy’ scales. Public Health Nutrition 2014, 17, 877–883, doi:10.1017/S1368980013000530. (p. 887)
“Nutrition literacy focuses mainly on abilities to understand nutrition information, which can be seen as a prerequisite for a wider range of skills described under the term food literacy. Thus, nutrition literacy can be seen a subset of food literacy.”	Krause, C.; Sommerhalder, K.; Beer-Borst, S.; Abel, T. Just a subtle difference? Findings from a systematic review on definitions of nutrition literacy and food literacy. Health Promotion International 2016, 33, 378–389, doi:10.1093/heapro/daw084. (p. 387)
**Food Literacy Definition**	**Reference**
“We defined food literacy as the capacity of an individual to obtain, interpret and understand basic food and nutrition information and services and the competence to use that information and services in ways that are health-enhancing. This definition was derived from the accepted definition of health literacy…”	Kolasa, K.M.; Peery, A.; Harris, N.G.; Shovelin, K. Food Literacy Partners Program: A Strategy To Increase Community Food Literacy. Topics in Clinical Nutrition 2001, 16, 1–10. (p. 5)
“…food literacy as more than knowledge; it also involves the motivation to apply nutrition information to food choices. Whereas food knowledge is the possession of food-related information, food literacy entails both understanding nutrition information and acting on that knowledge in ways consistent with promoting nutrition goals and “food well-being”.”	Block, L.G.; Grier, S.A.; Childers, T.L.; Davis, B.; Ebert, J.E.J.; Kumanyika, S.; Laczniak, R.N.; Machin, J.E.; Motley, C.M.; Peracchio, L.; et al. From nutrients to nurturance: A conceptual introduction to food well-being. Journal of Public Policy and Marketing 2011, 30, 5–13, doi:10.1509/jppm.30.1.5. (p. 7)
“…expands traditional measures of nutrition knowledge to include not only what people know about food but their ability to use that information to facilitate higher levels of food well-being. Food literacy ranges from declarative types of knowledge (e.g., knowing what asparagus is and what types of the nutrients asparagus might provide) to procedural knowledge (e.g., how to cook this vegetable).”	Bublitz, M.G.; Peracchio, L.A.; Andreasen, A.R.; Kees, J.; Kidwell, B.; Miller, E.G.; Motley, C.M.; Peter, P.C.; Rajagopal, P.; Scott, M.L. The quest for eating right: Advancing food well-being. Journal of Research for Consumers 2011, 1. (pp. 3–4)
“Food literacy was seen mainly as an individual’s ability to read, understand, and act upon labels on fresh, frozen, canned, frozen, processed, and takeout food.”	Fordyce-Voorham, S. Identification of essential food skills for skill-based healthful eating programs in secondary schools. J Nutr Educ Behav 2011, 43, 116–122, doi:10.1016/j.jneb.2009.12.002. (p. 119)
“I take the inspiration for food literacy from the notions of ‘health literacy’ in public health literature. The concept was born out of public health policy’s endeavor to educate people to seek healthier lifestyles and adhere to prescribed advice and was used to explain ‘the relationship between the patient literacy levels and their ability to comply with prescribed therapeutic regimens’.”	Kimura, A.H. Food education as food literacy: Privatized and gendered food knowledge in contemporary Japan. Agriculture and Human Values 2011, 28, 465–482, doi:10.1007/s10460-010-9286-6. (p. 479)
“Food literacy is the ‘capacity of an individual to obtain, interpret and understand basic food and nutrition information and services as well as the competence to use that information and available services that are health enhancing.’”	Pendergast, D.; Garvis, S.; Kanasa, H. Insight from the Public on Home Economics and Formal Food Literacy. Family and Consumer Sciences Research Journal 2011, 39, 415–430, doi:10.1111/j.1552-3934.2011.02079.x. (p. 418)
“…the relative ability to basically understand the nature of food and how it is important to you, and how able you are to gain information about food, process it, analyze it and act upon it.”	Vidgen, H.A.; Gallegos, D. What is food literacy and does it influence what we eat: a study of Australian food experts. 2011. (p. ii)
“… a complex, interrelated, person-centred set of skills that are necessary to provide and prepare safe, nutritious, and culturally-acceptable meals for all members of one’s household.”	Thomas, H.M.; Irwin, J.D. Cook It Up! A community-based cooking program for at-risk youth: Overview of a food literacy intervention. BMC Research Notes 2011, 4, 495, doi:10.1186/1756-0500-4-495. (p. 6)
“A collection of inter-related knowledge, skills and behaviours required to plan, manage, select, prepare and eat foods to meet needs and determine food intake. Food literacy is the scaffolding that empowers individuals, households, communities or nations to protect diet quality through change and support dietary resilience over time.”	Vidgen, H.A.; Gallegos, D. Defining food literacy, its components, development and relationship to food intake: A case study of young people and disadvantage. 2012. (p. vii)
“…the capacity of an individual to obtain, process and understand basic food information about food and nutrition as well as the competence to use that information in order to make appropriate health decisions.”	Murimi, M.W. Healthy literacy, nutrition education, and food literacy. Journal of Nutrition Education and Behavior 2013, 45, 195, doi:10.1016/j.jneb.2013.03.014. (p. 195)
“…focuses on food and nutrition information to help individuals make appropriate eating decisions.”	Rawl, R.; Kolasa, K.M.; Lee, J.; Whetstone, L.M. A Learn and Serve Nutrition Program: The Food Literacy Partners Program. Journal of Nutrition Education and Behavior 2008, 40, 49–51, doi:10.1016/j.jneb.2007.04.372. (p. 49)
“…a set of skills and attributes that help people sustain the daily preparation of healthy, tasty, affordable meals for themselves and their families. Food literacy builds resilience, because it includes food skills (techniques, knowledge and planning ability), the confidence to improvise and problem-solve, and the ability to access and share information. Food literacy is made possible through external support with healthy food access and living conditions, broad learning opportunities, and positive socio-cultural environments.”	Desjardins, E. Making Something out of Nothing: Food Literacy Among Youth, Young Pregnant Women and Young Parents Who are at Risk for Poor Health. A Locally Driven Collaborative Project. 2013. At: http://www.osnpph.on.ca/upload/membership/document/foodliteracy-study.ldcpontario.final.dec2013.pdf (accessed on 23 January 2023) (p. 70)
“Food literacy can be defined as an individual’s food related knowledge, attitudes, and skills. This broad definition of food literacy incorporates household perception, assessment, and management of the risks associated with their food choices. Individuals’ food literacy level influences their food-related decisions, which ultimately impact their diet and health as well as the environment.”	Howard, A.; Brichta, J. What’s to Eat?: Improving Food Literacy in Canada. 2013. (p. 2)
“Food literacy is the ability to “read the world” in terms of food, thereby recreating it and remaking ourselves. It involves a full-cycle understanding of food—where it is grown, how it is produced, who benefits and who loses when it is purchased, who can access it (and who can’t), and where it goes when we are finished with it. It includes an appreciation of the cultural significance of food, the capacity to prepare healthy meals and make healthy decisions, and the recognition of the environmental, social, economic, cultural, and political implications of those decisions.”	Sumner, J. Food literacy and adult education: Learning to read the world by eating. Canadian Journal for the Study of Adult Education 2013, 25, 79–92. (p. 86)
“Functional food literacy: basic communication of credible, evidence-based food and nutrition information, involving accessing, understanding and evaluating information. Interactive food literacy: development of personal skills regarding food and nutrition issues, involving decision making, goal setting and practices to enhance nutritional health and well-being Critical food literacy: respecting different cultural, family and religious beliefs in respect to food and nutrition (including nutritional health), understanding the wider context of food production and nutritional health, and advocating for personal, family and community changes that enhance nutritional health.”	Slater, J. Is cooking dead? The state of Home Economics Food and Nutrition education in a Canadian province. International Journal of Consumer Studies 2013, 37, 617–624, doi:10.1111/ijcs.12042. (p. 623)
“Food literacy is the scaffolding that empowers individuals, households, communities or nations to protect diet quality through change and strengthen dietary resilience over time. It is composed of a collection of inter-related knowledge, skills and behaviours required to plan, manage, select, prepare and eat food to meet needs and determine intake.”	Vidgen, H.A.; Gallegos, D. Defining food literacy and its components. Appetite 2014, 76, 50–59, doi:10.1016/j.appet.2014.01.010. (p. 54)
“Food literacy is the ability of an individual to understand food in a way that they develop a positive relationship with it, including food skills and practices across the lifespan in order to navigate, engage, and participate within a complex food system. It’s the ability to make decisions to support the achievement of personal health and a sustainable food system considering environmental, social, economic, cultural, and political components.”	Cullen, T.; Hatch, J.; Martin, W.; Higgins, J.W.; Sheppard, R. Food literacy: Definition and framework for action. Canadian Journal of Dietetic Practice and Research 2015, 76, 140–145, doi:10.3148/cjdpr-2015-010. (p. 143)
“We suggest using the term food literacy instead of nutrition literacy to describe the wide range of skills needed for a healthy and responsible nutrition behaviour. When measuring food literacy, we suggest the following core abilities and skills be taken into account: reading, understanding, and judging the quality of information; gathering and exchanging knowledge related to food and nutrition themes; practical skills like shopping and preparing food; and critically reflecting on factors that influence personal choices about food, and understanding the impact of those choices on society.”	Krause, C.; Sommerhalder, K.; Beer-Borst, S.; Abel, T. Just a subtle difference? Findings from a systematic review on definitions of nutrition literacy and food literacy. Health Promotion International 2016, 33, 378–389, doi:10.1093/heapro/daw084. (p. 387)
“‘food literacy’ encompasses a more holistic approach to describe the practicalities needed to meet nutrition recommendations: plan, management, selection, preparation, and consumption.”	Garcia, A.L.; Reardon, R.; McDonald, M.; Vargas-Garcia, E.J. Community Interventions to Improve Cooking Skills and Their Effects on Confidence and Eating Behaviour. Current Nutrition Reports 2016, 5, 315–322, doi:10.1007/s13668-016-0185-3. (p. 316)
“…food literacy is a complex phenomenon made up of multiple attributes, including those that are both intrinsic and extrinsic. By conceptualizing these attributes, the results of the present scoping review provide the foundation for the development of a measurement tool that can support monitoring and the evaluation of interventions to support food literacy.”	Perry, E.A.; Thomas, H.; Samra, H.R.; Edmonstone, S.; Davidson, L.; Faulkner, A.; Petermann, L.; Manafò, E.; Kirkpatrick, S.I. Identifying attributes of food literacy: A scoping review. Public Health Nutrition 2017, 20, 2406–2415, doi:10.1017/S1368980017001276. (p. 2413)
“Food Literacy (FL) is the combination of knowledge, skills, and behaviours required to plan, select, manage, prepare, and consume foods that meet nutritional recommendations. Understood to be an important component of healthy living, FL is associated with confidence, autonomy, and empowerment towards food.”	Bomfim, M.C.C.; Wallace, J.R. Pirate bri’s grocery adventure: Teaching food literacy through shopping. In Proceedings of the Conference on Human Factors in Computing Systems—Proceedings, 2018., (p. 2)

## Data Availability

Not applicable.
